# Screening for Prostate Cancer: The Debate Continues

**DOI:** 10.6004/jadpro.2013.4.1.2

**Published:** 2013-01-01

**Authors:** Marc R. Matrana, Bradley Atkinson

**Affiliations:** From University of Texas MD Anderson Cancer Center, Houston, Texas

## Abstract

Other than skin cancer, prostate cancer is the most frequently diagnosed cancer in men, and it is the second leading cause of cancer-related death for males in the United States. Screening for prostate cancer using prostate-specific antigen testing became widely used by the late 1980s, augmenting the digital rectal exam. This led to a decline in the percentage of prostate cancer cases that were metastatic at diagnosis and a decrease in prostate cancer mortality. But some argued it led to overtreatment of prostate cancers as well. Recently, the U.S. Preventive Services Task Force (USPSTF) issued recommendations against routine prostate cancer screening in asymptomatic patients. The recent recommendations have created much controversy among medical professionals, patient advocate groups, and the general public. Most prostate cancer screening recommendations from professional organizations agree that an informed discussion and review of each individual patient’s clinical situation should drive the decision to screen or not to screen, but the current USPSTF recommendations largely remove patient and provider autonomy in this regard. They do not contribute toward personalized screening based on individualized patient risk profiles, characteristics, and preferences.

In 2012, the United States Preventive Services Task Force (USPSTF) issued recommendations against routine prostate cancer screening in asymptomatic male patients regardless of age, race, ethnicity, or family history (USPSTF, 2012). Previous USPSTF guidelines had recommended against screening in men 75 years or older and noted that "evidence is insufficient to assess the balance of benefits and harms of prostate cancer screening in men younger than age 75 years." The task force now concludes that the potential harms of screening for prostate cancer outweigh the benefits.

These recommendations join a larger set of screening recommendations from the USPSTF that have often differed significantly from those of professional medical associations and cancer advocacy organizations. These new recommendations represent only the latest controversy in the debate about prostate cancer screening that has continued for decades.

## Background

Prostate cancer is the most frequently diagnosed cancer in men (other than skin cancer), and it is the second leading cause of cancer-related deaths in males in the United Sates. It is estimated that in 2012, 241,740 men were diagnosed with prostate cancer and 28,170 patients died of the disease in the United States (Siegel, Naishadham, & Jemal, 2012). Advanced age, family history, and African ancestry are all associated with a greater risk of prostate cancer. Approximately 90% of prostate cancers diagnosed in the United States are initially detected via screening (Hoffman, Stone, Espey, & Potosky, 2005). Many of these cancers may be indolent, and overtreatment due to aggressive screening is of great concern. Autopsy studies suggest that 30% of men older than 50 years and 70% of men older than 70 years have occult prostate cancer, yet most die of other causes (Coley, Barry, Fleming, Fahs, & Mulley, 1997; Coley, Barry, Fleming, & Mulley, 1997).

Prostate-specific antigen (PSA) testing was initially developed for prostate cancer surveillance, but by the late 1980s it became widely used for screening, augmenting the digital rectal exam (DRE). By 2001, 75% of men 50 years or older in the United States had undergone PSA testing (Sirovich, Schwartz, & Woloshin, 2003). As screening with PSA became common, the percentage of prostate cancer cases that were metastatic at diagnosis declined from 25% in 1980 to 4% in 2002 (Etzioni, Gulati, Falcon, & Penson, 2008). Prostate cancer mortality also decreased by 4.1% annually between 1994 and 2006, a change thought to be due in large part to PSA screening as there were minimal clinical treatment advances during that time period (Jemal, Siegel, Xu, & Ward, 2010).

Current recommendations from US health organizations vary (see Table 1), but most agree that an informed discussion of the risk vs. benefits of screening should precede any actual screening. The American Cancer Society (ACS) recommends that discussions begin at age 50 in men with a life expectancy over 10 years, at age 45 in men at high risk (African Americans or those with a first-degree relative diagnosed at age < 65), or at age 40 in men with the highest risk (multiple first-degree relatives with prostate cancer; Smith et al., 2011; Wolf et al., 2010).

**Table 1:**
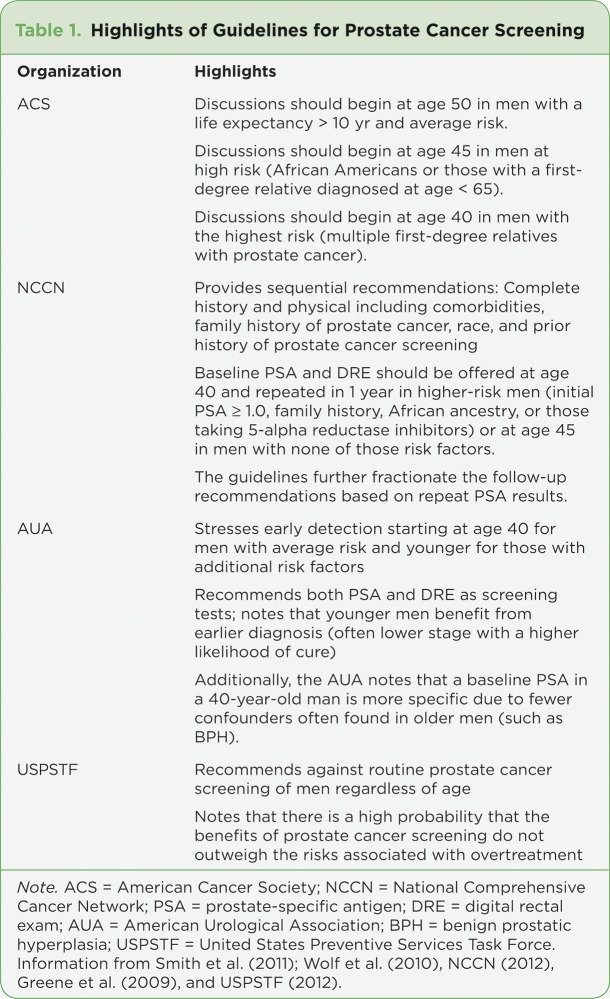
Table 1. Highlights of Guidelines for Prostate Cancer Screening

The National Comprehensive Cancer Network (NCCN) guidelines provide a set of sequential recommendations that recommend a complete history and physical exam with questions regarding comorbidities, family history of prostate cancer, race, and prior history of prostate cancer screening (NCCN, 2012). Baseline PSA and DRE should be offered at age 40 and repeated a year later in higher-risk men (those with an initial PSA ≥ 1.0, family history, African ancestry, or those taking 5-alpha reductase inhibitors) or at age 45 in men with none of the above-mentioned risk factors. The guidelines further fractionate the follow-up recommendations based on repeat PSA results.

The American Urological Association (AUA) guidelines stress early detection starting at 40 years old for men with average risk and younger for those with additional risk factors. The AUA guidelines recommend both PSA and DRE as screening tests (Greene et al., 2009). While the AUA acknowledges that prostate cancer in men younger than 50 is uncommon, the group argues that younger men benefit from earlier diagnosis as their cancer often represents lower-stage disease with a higher likelihood of cure. Additionally, the AUA notes that a baseline PSA in a 40-year-old man is more specific due to fewer confounders, such as benign prostatic hyperplasia, than often found in older men (Carter, Epstein, & Partin, 1999; Khan, Han, Partin, Epstein, & Walsh, 2003).

## Latest Controversy and Current Data

In May 2012, the USPSTF published guidelines regarding prostate cancer screening (USPSTF, 2012). The task force recommends against routine prostate cancer screening of men regardless of age, noting that there is a high probability that the benefits of prostate cancer screening do not outweigh the risks associated with overtreatment. The recent recommendations have created much controversy among medical professionals, patient advocate groups, and the general public.

The new USPSTF recommendations are based on an extensive review of the currently available evidence, although some have been critical of the conclusions drawn from this review. Criticism includes the fact that the USPSTF recommendations relied heavily on a meta-analysis by Chou et al. (2011a; 2011b) that weighted equally trials of varying quality (Schröder et al., 2009). The two largest randomized controlled trials of prostate cancer screening included in the meta-analysis were the US-based Prostate, Lung, Colorectal, and Ovarian (PLCO) cancer screening trial and the European Randomized study of Screening for Prostate Cancer (ERSPC).

## THE PLCO TRIAL

The PLCO trial included 76,693 men who were randomized to screening vs. control arms (Andriole et al., 2012). Patients assigned to the screening group were offered annual PSA testing for 6 years and annual DRE for 4 years, whereas those in the control group were not actively screened but sometimes received screening outside of the study. Recently, extended follow-up to 13 years after the trial has been reported. Approximately 92% of the study participants were followed to 10 years and 57% to 13 years. At 13 years, 4,250 men had been diagnosed with prostate cancer in the screening arm compared with 3,815 in the control arm. Incidence rates for prostate cancer in the screening and control arms were 108.4 and 97.1 per 10,000 person-years, respectively, resulting in a relative increase of 12% in the screening arm (RR = 1.12, 95% confidence interval [CI] = 1.07–1.17). The mortality rates from prostate cancer in the screening and control arms were 3.7 and 3.4 deaths per 10,000 person-years, respectively (RR = 1.09, 95% CI = 0.87–1.36). No statistically significant interactions with respect to prostate cancer mortality and age, pretrial PSA testing, and comorbidity were observed between trial arms. The clinical cancer stages and treatment distribution were similar across trial arms.

While the PLCO trial was generally well designed, it has notable limitations. Particularly concerning was the extent of opportunistic PSA screening that occurred in the control arm. Approximately 45% of patients had at least one PSA test in the 3 years prior to randomization, and more than 50% of participants in the control arm had PSA screening by the sixth year of the trial. In addition, of the men in the screening arm, only 40% of participants who had an abnormal initial PSA value actually underwent a prostate biopsy, and those proportions were lower with subsequent screening. Lastly, a PSA level of 4 ng/mL was used to initiate further workup. A lower cutoff value may lead to detection of more cancers with lower clinical stage and better cancer-specific survival (Andriole et al., 2012).

## THE ERSPC TRIAL

The ERSPC trial was a multi-institutional randomized controlled trial that accrued 182,000 men between the ages of 50 and 74 (Schröder et al., 2009). Participants in this trial were randomized to receive PSA screening every 2 to 7 years in the screening group or to the control group who were not screened. Approximately 87% of the screening participants received PSA screening every 4 years, and most centers used a PSA cutoff of 3 ng/mL to initiate biopsy. At a median follow-up of 9 years, the incidence of prostate cancer was 8.2% in the screening group and 4.8% in the control group. The rate ratio for death from prostate cancer in the screening group compared with the control group, was 0.80 (95% CI = 0.65–0.98; adjusted * p* = .04), thereby a 20% relative risk reduction in mortality was noted in the screening group.

In contrast to the PLCO trial, opportunistic PSA screening in the control group was not common (about 20%). However, limitations of the ERSPC trial include inconsistent screening intervals and PSA cutoffs among study centers, screening group participants were more likely to receive treatment at academic centers at diagnosis, and the relatively short median follow-up of 9 years likely underestimates the survival benefit. Lastly, the risk of overdiagnosis in the ERSPC trial has been estimated to approach 50% while the benefit of survival was restricted to patients in the core age group of 55 to 69 at randomization.

## GÖTEBORG CENTER DATA

The Göteborg Center, a single center from within the multicenter ERSPC trial, reported its data after the ERSPC was published. The trial was designed and initiated independently from the ERSPC, although it subsequently agreed to include a subset of participants in the ERSPC (Hugosson et al., 2010). In the Göteborg trial, 20,000 men between the ages of 50 and 64 were randomized to undergo PSA screening every 2 years vs. usual care. During a median follow-up of 14 years, the incidence of prostate cancer in the screening arm was 12.7% compared to 8.2% in the control arm (hazard ratio, 1.64; 95% CI = 1.50–1.80; * p* <.0001). The absolute risk reduction of death from prostate cancer at 14 years was 0.40% (95% CI = 0.17–0.64), from 0.90% in the control group to 0.50% in the screening group. Subjects in the screening arm were diagnosed more frequently with lower-stage disease and had a lower incidence of metastases at the time of diagnosis.

The findings of the Göteborg study demonstrated better outcomes than either the ERSPC or PLCO trials. The Göteborg trial design included several elements that may account for these positive findings. The trial enrolled younger patients with a median age of 56 years compared to the ERSPC and PLCO studies, which both enrolled patient populations with a median age of > 60 years. Additionally, men were screened more frequently with a PSA test every 2 years compared to every 4 years in the ERSPC study. The Göteborg trial had much lower rates of PSA testing prior to subject entry with approximately 3% of participants who had been previously screened compared to a rate of 44% in the PLCO study. Contamination rates for opportunistic PSA screening in the control group were also significantly lower. Lastly, the median follow-up duration was longer than reported in either the PLCO or ERSPC. The strengths of the Göteborg trial influence the positive findings and suggest that prostate cancer screening can have a positive impact on prostate cancer mortality.

Interpretation of the currently available data varies, especially with regard to the impact of factors such as contamination, follow-up duration, and statistical analysis. Gomella et al. (2011) argue that the level of evidence in favor of PSA screening for prostate cancer provided by the ERSPC and Göteborg trial is similar to that which has led to widespread screening for breast and colon cancers. A meta-analysis of breast cancer data demonstrated that a number needed to screen (NNS) to prevent 1 cancer-related death was 377 women aged 60 to 69 years and 1,339 women aged 50 to 59 years (Nelson et al., 2009). Likewise, a colorectal cancer screening trial with flexible sigmoidoscopy reported a NNS of 489 participants (Atkin et al., 2010). Comparably, the ERSPC demonstrated a NNS of 1,410 men while the Göteborg trial reported a NNS of 293 men for prostate cancer screening.

While some data from major trials appear to support screening, risks associated with widespread screening exist. Aggressive screening for prostate cancer leads to increased and sometimes unnecessary biopsies, which may be associated with infection, fever, bleeding, pain, and sexual and urinary symptoms. Early detection of prostate cancer may also lead to overtreatment of an otherwise indolent disease. Radical prostatectomy is associated with significant urinary and sexual dysfunction, as well as other complications including infection and death in the most extreme cases (Stanford et al., 2000; Salomon et al., 2002). In preliminary results of the Prostate cancer Intervention Versus Observation Trial (PIVOT) presented at the American Urological Association’s annual meeting in May 2011, early radical prostatectomy did not reduce mortality compared to observation in men with localized prostate cancer (Wilt et al., 2009; Wilt, 2011).

 Similar studies have reported comparable conclusions (Hardie et al., 2005; Klotz & Nam, 2006; Zietman, Thakral, Wilson, & Schellhammer, 2001). Surgery and radiation therapy for prostate cancer are associated with significant morbidity and mortality and should not be undertaken lightly. However, current technologies limit the extent to which clinicians can identify early indolent prostate cancer vs. those that will behave aggressively, leading most patients and practitioners to treat identified cancers aggressively (Welch & Albertsen, 2009).

## Conclusions

A dominant vision of most major cancer centers and professional organizations is a shift from "one size fits all" cancer therapies toward more precise, targeted, and individualized treatments tailored to the patient’s specific biology and pathophysiology. Likewise, it seems inevitable that as the underlying drivers of disease are better understood and the risk factors that predispose patients to certain malignancies are identified, the blanket "one size fits all" screening recommendations will also go out of favor. Although prostate cancer screening recommendations from professional organizations differ, they all agree that an informed discussion and review of each individual patient’s situation should drive the decision to screen or not to screen (McNaughton-Collins & Barry, 2011). The USPSTF draft recommendations largely remove patient and provider autonomy in this regard and do not contribute toward personalized screening based on individualized patient risk profiles, characteristics, and preferences. Until more precise and improved screening and diagnostic modalities are discovered and approved, identifying specific high-risk patients for PSA screening remains one of the paramount tools available in the fight against prostate cancer.
